# Downregulation of ATXN3 Enhances the Sensitivity to AKT Inhibitors (Perifosine or MK-2206), but Decreases the Sensitivity to Chemotherapeutic Drugs (Etoposide or Cisplatin) in Neuroblastoma Cells

**DOI:** 10.3389/fonc.2021.686898

**Published:** 2021-07-12

**Authors:** Baocheng Gong, Jinhua Zhang, Zhongyan Hua, Zhihui Liu, Carol J. Thiele, Zhijie Li

**Affiliations:** ^1^ Department of Pediatrics, Shengjing Hospital of China Medical University, Shenyang, China; ^2^ Medical Research Center, Liaoning Key Laboratory of Research and Application of Animal Models for Environment and Metabolic Diseases, Shengjing Hospital of China Medical University, Shenyang, China; ^3^ Cellular and Molecular Biology Section, Pediatric Oncology Branch, National Cancer Institute, National Institutes of Health, Bethesda, MD, United States

**Keywords:** neuroblastoma, ataxin-3, BIM, Bcl-xl, perifosine, MK-2206, etoposide, cisplatin

## Abstract

**Background:**

Chemotherapy resistance is the major cause of failure in neuroblastoma (NB) treatment. ATXN3 has been linked to various types of cancer and neurodegenerative diseases; however, its roles in NB have not been established. The aim of our study was to explore the role of ATXN3 in the cell death induced by AKT inhibitor (perifosine or MK-2206) or chemotherapy drugs (etoposide or cisplatin) in NB cells.

**Methods:**

The expressions of ATXN3 and BCL-2 family members were detected by Western blot. Cell survival was evaluated by CCK8, cell confluence was measured by IncuCyte, and apoptosis was detected by flow cytometry. AS and BE2 were treated with AKT inhibitors or chemotherapeutics, respectively.

**Results:**

Downregulation of ATXN3 did not block, but significantly increased the perifosine/MK-2206-induced cell death. Among the BCL-2 family members, the expression of pro-apoptotic protein BIM and anti-proapoptotic protein Bcl-xl expression increased significantly when ATXN3 was down-regulated. Downregulation of BIM protected NB cells from the combination of perifosine/MK-2206 and ATXN3 downregulation. Downregulation of ATXN3 did not increase, but decrease the sensitivity of NB cells to etoposide/cisplatin, and knockdown of Bcl-xl attenuated this decrease in sensitivity.

**Conclusion:**

Downregulation of ATXN3 enhanced AKT inhibitors (perifosine or MK-2206) induced cell death by BIM, but decreased the cell death induced by chemotherapeutic drugs (etoposide or cisplatin) *via* Bcl-xl. The expression of ATXN3 may be an indicator in selecting different treatment regimen.

## Introduction

Neuroblastoma (NB) derives from developing sympathetic nervous system and is the most common solid pediatric tumors in children. Although there are only 25 to 50 cases per million, it still accounts for almost 15% of pediatric cancer-related death in children (1). According to the age at diagnosis, MYCN amplification status and genomic characterization, pathogenic type, NB patients were divided into low-risk, intermediate-risk, and high-risk groups. Patients with low risk always show excellent outcomes; however, about half of all the NB patients are classified as high-risk group, which is associated with a poor prognosis (2). Despite recent advances in immunotherapy and targeted therapy for NB, the 5-year overall survival (OS) rate of the high-risk group is still less than 40% (1). Chemotherapy is the most common method for NB patients in the clinic, but chemotherapy drug resistance is the leading cause of failure in NB treatment, highlighting the need for exploring potential therapeutic targets of drug sensitivity and new strategies for NB patients.

AKT was first discovered as an oncogene in leukemia (3), it regulates the proliferation, metabolism, angiogenesis, migration, and invasion of cancer cells (4, 5). Aberrant activation of AKT was found in many adult tumors, such as multiple myeloma (6, 7), renal cancer (8), lung cancer (9), prostate cancer (10), and liver cancer (11). Hyperactivation of AKT has been explored as a new poor prognostic factor for NB patients and is significantly correlated with MYCN amplification and advanced stage (12). We have previously reported that AKT mediates the BDNF/TrkB-induced chemoresistance, and activation of AKT could promote the proliferation of NB cells (13). Treatment targeting AKT provides us an enormous potential to improve the prognosis of NB. To date, many types of AKT inhibitors have been investigated, such as phosphatidylinositol analog inhibitors, ATP-competitive inhibitors, allosteric AKT kinase inhibitors, and alkyl phospholipids (14, 15). Perifosine, an alkyl-phospholipid AKT, shows low toxicity and high bioavailability. It could block the translocation of AKT to plasma membrane and the subsequent phosphorylation (16). The phase II clinical trials of perifosine showed great potential in anti-tumors, furthermore, we have found that perifosine exerts a satisfactory antitumor effect in NB cells *in vivo* and *in vitro* (17–19). In a multicentral phase I clinical trial of peifosine for recurrent or refractory neuroblastoma, perifosine showed well therapeutic effects (20). Besides, perifosine also participates in mitogen-activated protein kinase (MAPK) (21), nuclear factor-κB (22) and autography signal pathway (23), and targets epidermal growth factor receptor (EGFR) (24, 25) and death receptor (DR4/DR5) (26) to inhibit the growth of cancer cells. Accordingly, perifosine had multiple functions, beyond its role as an AKT inhibitor. Because of the great potential value for the treatment of NB, we performed proteome analysis by using SILAC labeling and LC-MS/MS analysis in perifosine treated AS cells (27), and ATXN3 was one of those genes whose expressions were increased significantly after perifosine treatment.

ATXN3, a member of deubiquitylates (DUBs), shows complex relationships with several substrates such as P53, Beclin1, Chk1, histone H2B, and HDAC (28–31). The DUBs family consists of about 90 enzymes that promote the removal of ubiquitin from proteins, the process of deubiquitylation plays a critical role in cellular homeostasis and the development of various tumors (32–34). ATXN3 also contributes to the development and progression of breast cancer (35), lung cancer (36), gastric cancer (32), colorectal cancer (37), and testicular cancer (38). In addition, ATXN3 has been identified as a potential therapeutic target for neurodegenerative diseases (39). However, studies on the role of ATXN3 in NB are lacking, and we hypothesized that ATXN3 mediates the function of perifosine for antitumor. In this study, we explored the role of ATXN3 in the cell death induced by AKT inhibitors (perifosine or MK-2206) or chemotherapy drugs (etoposide or cisplatin) in NB cells.

## Materials and Methods

### Cell Culture

SK-N-AS (AS) and SK-N-BE2 (BE2) used in this study were received from Dr. Carol J. Thiele (National Institutes of Health, USA). NB cells were cultured in RPMI 1640 media (Bionind, Israel) at 37°C in 5% CO_2_ incubator, which contained 10% fetal bovine serum (FBS) (Bionind, Israel), 2 mM glutamine, antibiotics penicillin (100 units/ml), and streptomycin (100 µg/ml) (Invitrogen, USA).

### Cell Transfection

The AS and BE2 cells were seeded into six-well plates in a density of 2 × 10^5^/well. JetPRIME reagent (Polyplus Transfection, Illkirsch, France) was used for the following siRNAs transfection. When the cell confluence was up to 60% to 70%, cells were washed with PBS twice, and incubated in 1640 media without penicillin or streptomycin. Then ATXN3 siRNA or BIM siRNAs were transfected into NB cells. Eight hours after the transfection, cells were cultured in new 1640 media for further treatment. The sequences of small interfering RNAs (ATXN3 siRNAs, BIM siRNAs, and control siRNA) (designed by Tongyong, Anhui, China) were as follows: ATXN3 siRNA#1: GGACAGAGUUCACAUCCAUCCAUTT; ATXN3 siRNA#2: GGACAGAGUUCACAUCCAUTT; ATXN3 siRNA#3: GCAAAAGCAGCAACAGCAGTT; BIM siRNA#1: CAACCTTCTGATGTAAGT; BIM siRNA#2CTACCTCCCTACAGACAGA; BIM siRNA#3: GTATTGGAGACGATTTAA; Bcl-xl siRNA#1: CUGUGAUACAAAAGAUCUUTT; Bcl-xl siRNA#2: CUUUCUCUCCCUUCAGAAUTT; Bcl-xl siRNA#3: CAUAUCAGAGCUUUGAACATT; Control siRNA: UUCUCCGAACGUGUCACGUTT.

### Cell Treatment

To explore the function of ATXN3 in AKT inhibitor (perifosine or MK-2206) and chemotherapeutics (etoposide or cisplatin), 16 h after the transfection, cells were seeded into 96-wells plates for 24 h, then treated with perifosine (AS, 7.5 μM; BE2, 10 μM), MK-2206 (AS, 10 μM; BE2, 7.5 μM), etoposide (AS, 1 μg/ml; BE2, 4 μg/ml) or cisplatin (AS, 1 μg/ml; BE2, 2 μg/ml) respectively, for 48 h.

### IncuCyte Zoom Live Imaging System and Cell Survival Analysis

AS and BE2 cells in 96-well plates under different conditions were incubated in IncuCyte zoom living cell imaging system (Essen Bioscience, USA) to evaluate the cell confluence for real time, and the images were obtained. Forty-eight hours after the treatment of these drugs, the real-time evaluation of cell confluence and Cell Counting Kit-8 (CCK8 assay) were used to evaluate the cell proliferation and cell survival. The CCK8 assay was performed as the manufacturer’s instruction. Absorbance was measured at 450 nm.

### Apoptosis Analysis

Sixteen hours after transfection, NB cells were treated with perifosine (AS, 7.5 μM; BE2, 10 μM), MK-2206 (AS, 10 μM; BE2, 7.5 μM), etoposide (AS, 1 μg/ml; BE2, 4 μg/ml), or cisplatin (AS, 1 μg/ml; BE2, 2 μg/ml), respectively, for 48 h. Apoptotic cells were detected by flow cytometry using Annexin V staining and PI staining. In detail, all cells in plate were collected, then washed with PBS once. Then, they were suspended in 1× Annexin V binding solution (Dojindo, Japan), diluted to 1 × 10^5^ cells/ml, and transferred to a new tube. Then 5 μl of Annexin V or PI staining solution or both were added to the EP tubes and incubated in dark conditions for 15 min at room temperature. Finally, Annexin V/PI flow cytometry was performed as the manufacturer’s instruction.

### Western Blot

The AS and BE2 cells were collected after different treatment conditions: treated with perifosine at different concentrations for 24 or 48 h after the transfection of ATXN3 or BIM siRNAs (#1, #2, #3, or control), or transfected with ATXN3 siRNAs (#2 or control) for 16 h followed by treatment with perifosine (AS, 7.5 μM; BE2, 10 μM) or MK-2206 (AS, 10 μM; BE2, 7.5 μM) for 24 h. Then, total protein was extracted by whole cell lysis assay kits (Keygen Biotech, China) following the manufacturer’s protocol. The protein (40 μg) was loaded into SDS-PAGE gels with 40 μg/gel, and protein was transferred to PVDF membrane and incubated with 5% nonfat milk to block nonspecific antibody binding for 2 h. Then these membranes were incubated at 4°C overnight with specific antibodies: anti-ATXN3 (Abcam, UK, 1:1000), anti-BIM (Abcam, UK, 1:1000), anti-BID (Abcam, UK, 1:1000), anti-BAX (Abcam, UK, 1:1000), anti-BAK (Abcam, UK, 1:1000), anti-BAD (Abcam, UK, 1:1000), anti-puma (Proteintech, USA, 1:5000), anti-BCL-XL (Abcam, UK, 1:1000), anti-MCL1 (Abcam, UK, 1:1000), anti-BCL2 (Abcam, UK, 1:1000), and anti-GAPDH (Proteintech, USA, 1:5000). Membranes were washed with Tris-buffered saline–Tween 20 (TBST) three times for 5 min each time, and incubated with the appropriate horseradish peroxidase–conjugated goat anti-rabbit or anti-mouse antibodies (Proteintech, USA, 1:5000) for 1 h at room temperature. Then, the anti-bodies were detected by the enhanced chemiluminescence reagents (Thermo Scientific, USA).

### Statistical Analysis

Statistical analysis was performed by GraphPad Prism 8 software. Also, we analyzed the mean ± SD of the independent experiments by Student’s *t* test. P value < 0.05 was considered as statistically significant.

## Results

### Downregulation of ATXN3 Promoted the Perifosine-Induced Cell Death in NB Cells

**Figure 1:**
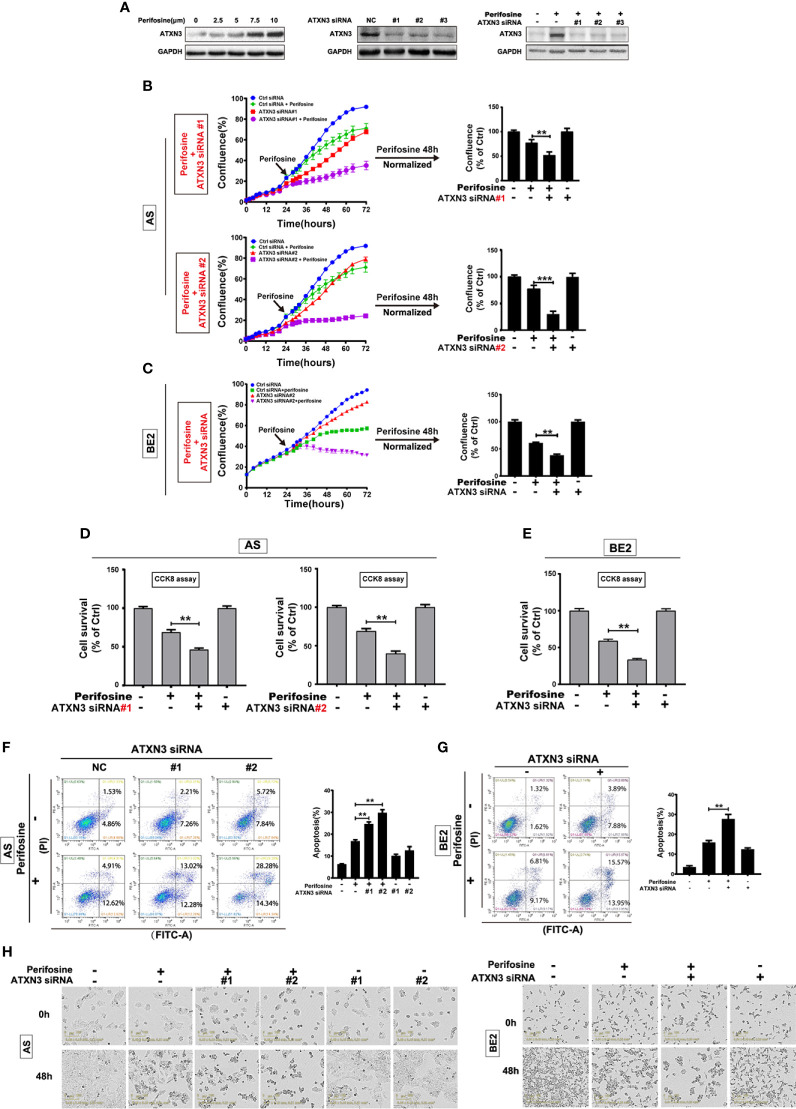
Downregulation of ATXN3 promoted perifosine-induced cell death in NB cells. AS cells were treated with different concentrations of perifosine (2.5, 5, 7.5, and 10 μM) for 24 h, or transfected with ATXN3 siRNA (#1, #2, #3, and control) for 48 h, or transfected with ATXN3 siRNA (#1, #2, and #3) for 16 h followed by 24 h treatment of perifosine. **(A)** The expression of ATXN3 were detected by Western blot; **(B, C)** AS cells were transfected with ATXN3 siRNAs (#1, #2), and BE2 cells were transfected with ATXN3 siRNA2 (marked as ATXN3 siRNA) for 16 h, followed by 48 h treatment of perifosine: Cell confluence was dynamically detected by IncuCyte Zoom and analyzed at the end of experiment; **(D, E)** Cell survival was detected by CCK8 assay; **(F, G)** cell apoptosis was detected by Annexin V/PI flow cytometry; **(H)** The images of AS and BE2 cells with ATXN3 siRNAs transfection and perifosine treatment at 0 h and 48 h were recorded. Bar, SD, **P < 0.01, ATXN3 siRNAs + perifosine *vs.* control siRNA + perifosine; ***P < 0.001, ATXN3 siRNA #2 + perfosine *vs.* control siRNA + perifosine.

In our previous proteomics study (27), we found that ATXN3 expression increased after perifosine treatment. To verify this, AS cells were treated with different concentrations of perifosine for 24 h, and whole cell lysates were collected for Western blotting analysis. **Figure 1A** (left) showed that perifosine induced a dose-dependent increase of ATXN3 at protein level, which was consistent to the proteomics result.

To explore the role of ATXN3 in perifosine-induced cell death in NB, we designed three ATXN3 siRNAs (#1, #2, #3), which were shown to down-regulate the endogenous ATXN3 expression (**Figure 1A**, middle) and perifosine-induced ATXN3 expression (**Figure 1A**, right). In the following studies, we transfected the ATXN3 siRNA #1 and #2 into AS cells, and transfected ATXN3 siRNA#2 (marked as ATXN3 siRNA) into BE2 cells. AS and BE2 cells transfected with control siRNA or ATXN3 siRNA were treated with perifosine for 48 h, and then cell confluence (**Figures 1B, C**), cell survival (**Figures 1D, E**), and cell apoptosis (**Figures 1F, G**) were evaluated by IncuCyte Zoom machine, CCK8 assay, and Annexin V/PI flow cytometry, respectively.

The cell confluence curves of ATXN3 siRNA (red curve)-transfected cells were slightly lower than that of the control siRNA-transfected cells (blue curve) (**Figures 1B, C**, left). When perifosine was added to the cells, the cell confluence curves of ATXN3 siRNA (purple curve)-transfected cells were significantly lower than that of the control siRNA-transfected cells (green curve). The confluence data of the cells treated with perifosine (48 h time point) were normalized by those of the cells without perifosine treatment in each control siRNA or ATXN3 siRNAs-transfected group, and then analyzed. As shown in **Figure 1B** (right side) and **Figure 1C** (right side), there was a significant decrease of cell confluence when ATXN3 expression was down-regulated (In AS cells, ATXN3 siRNA #1 and #2 *vs.* control siRNA: 51.9% and 30.6% *vs.* 77.5%, P < 0.01; In BE2 cells, ATXN3 siRNA *vs.* control siRNA: 38.1% *vs.* 60.8%, P < 0.01).

For the CCK8 assay, the survival rates of the cells transfected with siRNA for ATXN3 were significantly lower than that in control siRNA-transfected cells (in AS cells, ATXN3 siRNA #1 and #2 *vs.* control siRNA: 46.3% and 39.9% *vs.* 68.9%, P < 0.01, **Figure 1D**; in BE2 cells, ATXN3 siRNA *vs.* control siRNA: 33.6% *vs.* 59.2%, P < 0.01, **Figure 1E**).

For Annexin V/PI flow cytometry, under the condition of perifosine treatment, the apoptotic rate of NB cells with ATXN3 siRNAs transfected was significantly higher than control group (with perifosine treatment, in AS cells, ATXN3 siRNA #1 and #2 *vs.* control siRNA: 24.7% and 29.8% *vs.* 16.8%, P < 0.01, **Figure 1F**; in BE2 cells, ATXN3 siRNA *vs.* control siRNA: 27.7% *vs.* 15.9%, P < 0.01, **Figure 1G**). Besides, under microscope, there were more dead cells and less living cells when the cells were treated with a combination of ATXN3 downregulation and perifosine treatment compared with those treated with perifosine treated only (**Figure 1H**). These results suggested that downregulation of ATXN3 significantly enhanced perifosine-induced cell death in NB cells.

### BIM Mediated the Cell Death Induced by a Combination of Perifosine Treatment and ATXN3 Downregulation in NB Cells

**Figure 2:**
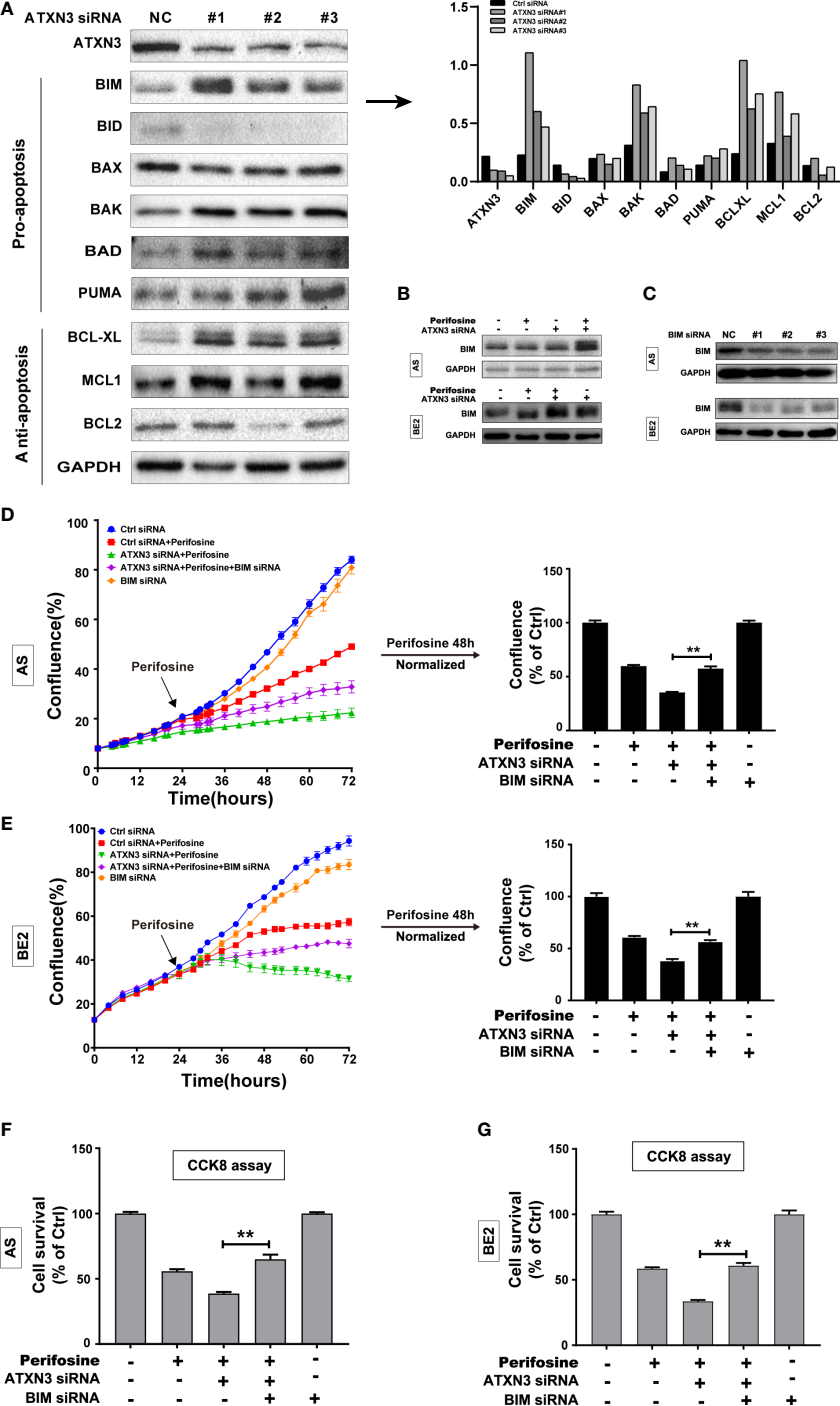
BIM mediated the cell death induced by a combination of perifosine treatment and ATXN3 downregulation in NB cells. **(A)** ATXN3 was down-regulated by ATXN3 siRNAs transfection in AS cells for 48 h, the expression of ATXN3 and BCL2 family members was detected by Western blot (left), and the protein quantitative analysis was performed (right); **(B)** AS and BE2 cells were transfected with ATXN3 siRNA #2 (marked as ATXN3 siRNA) for 16 h and treated with perifosine for 24 h, the expression of BIM was detected by Western blot; **(C)** AS and BE2 cells were transfected with BIM siRNAs (#1, #2, and #3) for 48 h, and the expression of BIM was detected by Western blot. BIM siRNA #1 (marked as BIM siRNA) and ATXN3 #2 (marked as ATXN3 siRNA) were transfected into AS and BE2 cells alone or combination, then these cells were treated with perifosine for 48 h; **(D, E)** The confluence was detected by IncuCyte Zoom; **(F, G)** Cell survival was detected by CCK8 assay. Bar, SD, **P < 0.01, ATXN3 siRNAs + perifosine + BIM siRNA *vs.* ATXN3 siRNA + perifosine.

To investigate the potential mechanism by which the downregulation of ATXN3-enhanced perifosine-induced cell death in NB cells, we knocked down ATXN3 by transfecting ATXN3 siRNA (control siRNA, #1, #2, and #3) into AS cells, and screened the expression changes of BCL-2 family members, then we performed protein quantitative analysis. As shown in **Figure 2A**, left, downregulation of ATXN3 increased the expressions level of pro-apoptotic member BIM, BAK, BAD, and anti-apoptotic member BCL-XL. Among them, BIM was the one whose expression increased the most significantly (**Figure 2A**, right). As shown in **Figure 2B**, a combination of ATXN3 downregulation and perifosine treatment furthermore increased the expression of BIM compared to ATXN3 downregulation only.

To explore whether BIM mediates the cell death induced by a combination of ATXN3 downregulation and perifosine treatment in NB cells, three BIM siRNAs (#1, #2, and #3) were designed, and all of them could down-regulate the expression of BIM (**Figure 2C**), and BIM siRNA #1 (marked as BIM siRNA) were used for further study. AS and BE2 cells were transfected with BIM siRNA #1 (marked as BIM siRNA) and ATXN3 siRNA #2 (marked as ATXN3 siRNA), followed by treatment with perifosine for 48 h. Then, cell confluence (**Figures 2D, E**) and cell survival (**Figures 2F, G**) were evaluated by IncuCyte Zoom machine and CCK8 assay, respectively. As shown in **Figures 2D, E** left, the confluence curve of the cells treated with perifosine + ATXN3 siRNA + BIM siRNA (purple curve) was significantly higher than that of the cells treated with perifosine+ATXN3 siRNA (green curve). At end of the experiment (48 h after perifosine treatment), there was a statistically significant increase of cell confluence in NB cells treated with perifosine + ATXN3 siRNA + BIM siRNA compared with cells treated with perifosine+ATXN3 siRNA (for perifosine treatment, ATXN3 siRNA + BIM siRNA *vs.* ATXN3 siRNA, in AS cells: 75.5% *vs.* 51.9%, P<0.01, **Figure 2D**, right; in BE2 cells: 56.6% *vs.* 38.1%, P<0.01, **Figure 2E**, right). The cell survival analysis detected by CCK8 assay showed that the survival rate of cells treated with perifosine + ATXN3 siRNA + BIM siRNA was also significantly higher than that of the cells treated with perifosine+ATXN3 siRNA (for perifosine treatment, ATXN3 siRNA + BIM siRNA *vs.* ATXN3 siRNA, in AS cells: 66.2% *vs.* 48.8%, P<0.01, **Figure 2F**; in BE2 cells: 60.8% *vs.* 33.6%, P<0.01, **Figure 2G**). All these data indicated that downregulation of BIM could block the cell death induced by a combination of ATXN3 downregulation and perifosine treatment.

### Downregulation of ATXN3 Enhanced MK-2206-Induced Cell Death by Upregulating BIM in NB Cells

**Figure 3:**
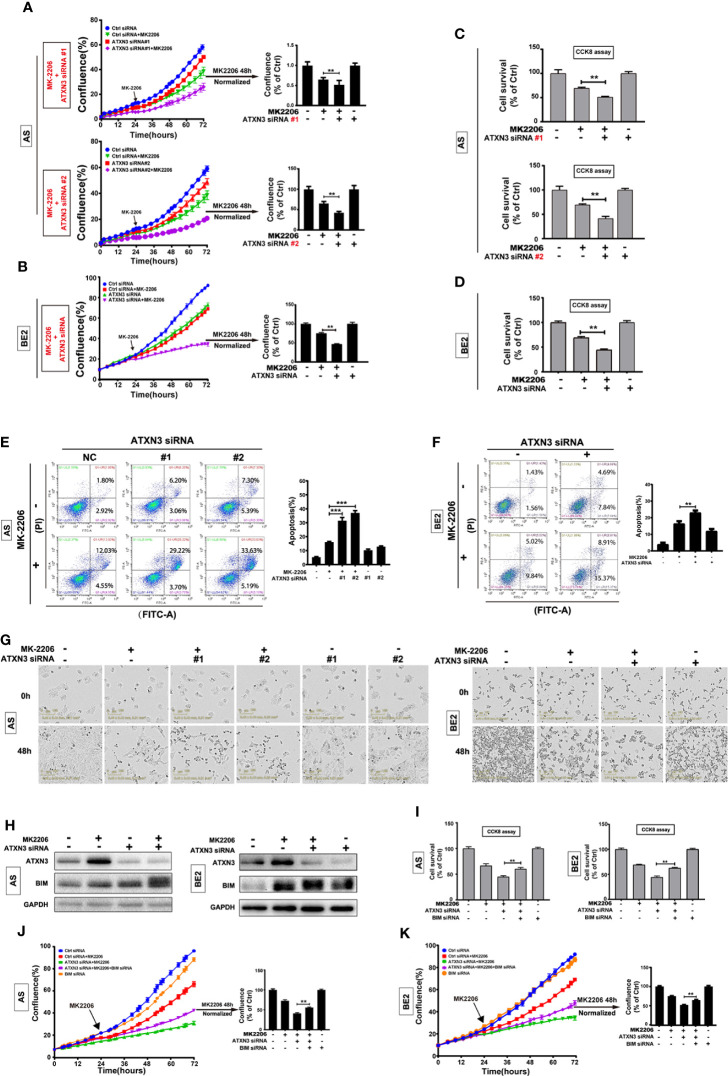
BIM mediated the cell death induced by a combination of MK-2206 treatment and ATXN3 downregulation in NB cells. AS cells were transfected with ATXN3 siRNAs (#1 and #2) and BE2 cells were transfected with ATXN3 siRNA #2 (marked as ATXN3 siRNA) for 16 h followed by 48 h treatment of MK-2206. **(A, B)** Cell confluence was dynamically detected by IncuCyte Zoom and analyzed at the end of experiment; **(C, D)** Cell survival was detected by CCK8 assay; **(E, F)** Cell apoptosis was detected by Annexin V/PI flow cytometry. Bar, SD, **, P< 0.01, ***, P<0.001, ATXN3 siRNAs + perifosine *vs.* control siRNA + perifosine; **(G)** The images of AS and BE2 cells with ATXN3 siRNAs transfection and perifosine treatment at 0 and 48 h were recorded; **(H)** AS and BE2 cells were transfected with ATXN3 siRNA2 (marked as ATXN3 siRNA) for 16 h, and treated with MK-2206 for 24 h, the expression of ATXN3 and BIM was detected by Western blot; **(I)** BIM siRNA #1 (marked as BIM siRNA) and ATXN3 siRNA #2 (marked as ATXN3 siRNA) were transfected into AS and BE2 cells alone or combination, then the cells were treated with MK-2206 for 48 h, cell survival was detected by CCK8 assay; **(J, K)** Cell confluence was detected by IncuCyte Zoom. Bar, SD, **, P<0.01, ATXN3 siRNA + MK-2206 + BIM siRNA *vs.* ATXN3 siRNA + MK-2206.

To investigate whether downregulation of ATXN3 could promote the cell death induced by other AKT inhibitors, we down-regulated the ATXN3 expression by transfecting ATXN3 siRNA into AS and BE2 cells and then treated the cells with an allosteric AKT inhibitor MK-2206. Cell confluence (**Figures 3A, B**), cell survival (**Figures 3C, D**), and cell apoptosis (**Figures 3E, F**) were evaluated by using similar assays described in **Figure 1**.

Under the condition of MK-2206 treatment, the confluence curves of the cells transfected with ATXN3 siRNA (purple curve) were significantly lower than that of the cells transfected with control siRNA (green curve) (**Figures 3A, B**, left). After 48 h for MK-2206 treatment, there was a statistically significant decrease of cell confluence in the cells transfected with ATXN3 siRNA (for MK-2206 treatment, in AS cells, ATXN3 siRNA #1 and #2 *vs.* control siRNA: 48.9% and 42.6% *vs.* 65.3%, P<0.01, **Figure 3A**, right; in BE2 cells, ATXN3 siRNA *vs.* control siRNA: 46.9% *vs.* 75.2%, P<0.01, **Figure 3B**, right).

The survival rates of the cells treated with MK-2206 in ATXN3 siRNA transfected cells are significantly lower than that of the cells transfected with control siRNA (for MK-2206 treatment, in AS cells, ATXN3 siRNA #1 and #2 *vs.* control siRNA: 51.2% and 41.5% *vs.* 69.5%, P<0.01, **Figure 3C**; in BE2 cells, ATXN3 siRNA *vs.* control siRNA: 44.5% *vs.* 69.3%, P<0.01, **Figure 3D**).

For Annexin V/PI flow cytometry analysis, under the condition of MK-2206 treatment, the apoptotic rate of NB cells transfected with ATXN3 siRNA was significantly higher than the control siRNA group (In AS cells, ATXN3 siRNA #1 and #2 *vs.* control siRNA: 37.0% and 31.4% *vs.* 16.1%, P<0.001 **Figure 3E**; In BE2 cells, ATXN3 siRNA *vs.* control siRNA: 23.0% *vs.* 16.3%, P<0.01, **Figure 3F**). Besides, under microscope, there were more dead cells and fewer living cells in the group treated with a combination of ATXN3 downregulation and MK-2206 compared to those cells treated with MK-2206 only (**Figure 3G**). These results suggested that downregulation of ATXN3 significantly enhanced MK-2206 induced cell death in NB cells.

To investigate whether BIM mediates the cell death induced by a combination of MK-2206 treatment and ATXN3 downregulation as it did with perifosine+ATXN3 downregulation, we first evaluated BIM expression under different conditions by Western blot. As shown in **Figure 3H**, MK-2206 treatment increased ATXN3 expression in AS cells, similar to the changes observed in cells treated with perifosine (**Figure 1A**). Either MK-2206 treatment or ATXN3 downregulation could increase the BIM expression, and the combination of MK-2206 treatment and ATXN3 downregulation increased the BIM expression to a higher level compared with each treatment alone (**Figure 3H**).

Then, we transfected ATXN3 siRNAs #2 (marked as ATXN3 siRNA) and BIM siRNA #1 (marked as BIM siRNA) into AS and BE2 cells, followed by treatment with MK-2206 for 48 h. The cell confluence and cell survival were analyzed. The survival rate of the cells treated with MK-2206 + ATXN3 siRNA + BIM siRNA was significantly higher than that of the cells treated with MK-2206 + ATXN3 siRNA (For MK-2206 treatment, ATXN3 siRNA + BIM siRNA *vs.* ATXN3 siRNA, in AS cells, 60.3% *vs.* 45.0%, P<0.01, **Figure 3I**, left; in BE2 cells: 62.7% *vs.* 44.5%, P<0.01, **Figure 3I**, right). The confluence curve of the cells treated with MK-2206+ATXN3 siRNA + BIM siRNA (purple curve) was higher than that of the cells treated with MK-2206+ATXN3 siRNA (green curve) (**Figures 3J, K**, left). After 48 h treatment of MK-2206, there was a statistically significant increase of cell confluence in cells treated with MK-2206 + ATXN3 siRNA + BIM siRNA compared with cells treated with MK-2206+ATXN3 siRNA (for MK-2206 treatment, ATXN3 siRNA + BIM siRNA *vs.* ATXN3 siRNA, in AS cells: 55.5% *vs.* 41.0%, P<0.01, **Figure 3J**, right; in BE2 cells: 64.8% *vs.* 52.6%, P<0.01, **Figure 3K**, right). All these data indicated that downregulation of BIM could partially block the cell death induced by a combination of MK-2206 treatment and ATXN3 downregulation.

### Downregulation of ATXN3 Decreased the Sensitivity of NB Cells to Etoposide

**Figure 4:**
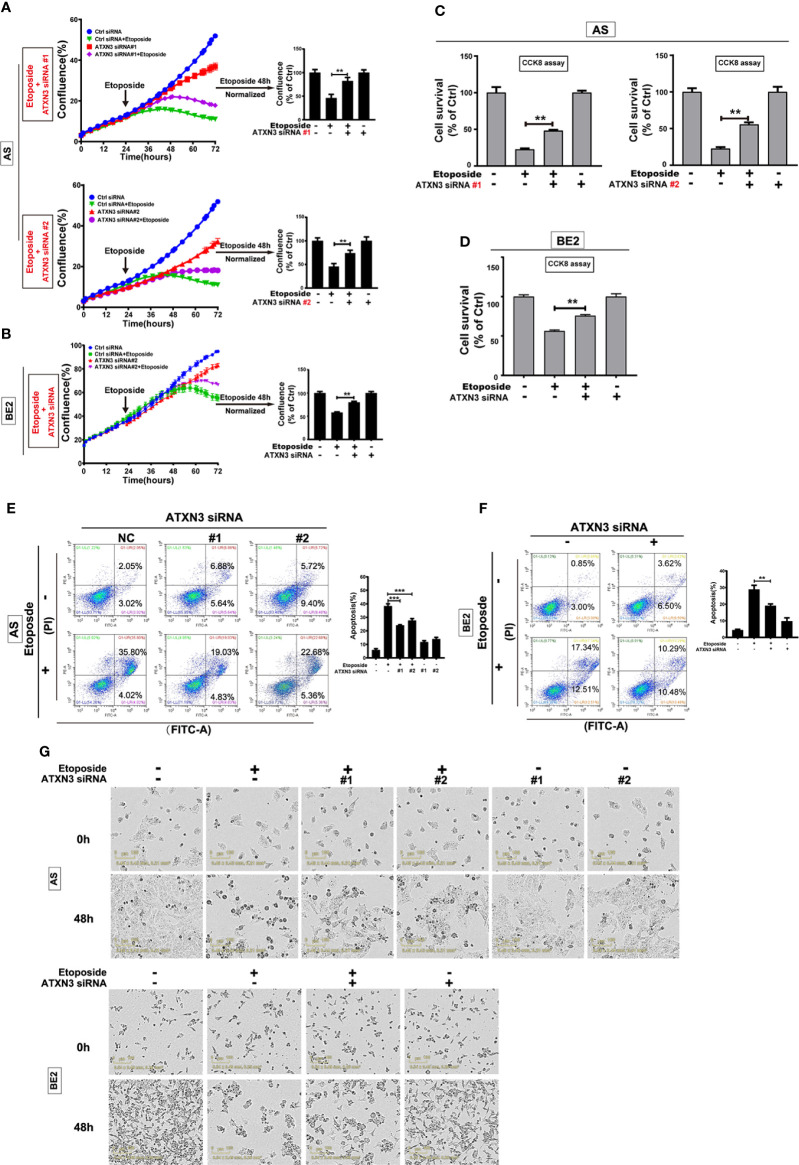
Downregulation of ATXN3 decreased the sensitivity of NB cells to etoposide. AS cells were transfected with ATXN3 siRNAs (#1, #2) and BE2 cells were transfected with ATXN3 siRNA #2 (marked as ATXN3 siRNA) for 16 h, followed by 48 h treatment of etoposide. **(A, B)** Cell confluence was dynamically detected and analyzed by IncuCyte Zoom; **(C, D)** Cell survival was detected by CCK8 assay; **(E, F)** Cell apoptosis was detected by Annexin V/PI flow cytometry; **(G)** The images of AS and BE2 cells with ATXN3 siRNAs transfection and etoposide treatment at 0 h and 48 h were recorded. Bar, SD, ***, P<0.001, **, P<0.01, ATXN3 siRNAs + etoposide *vs.* control siRNA + etoposide.

As we found that downregulation of ATXN3 promoted the cell death induced by AKT inhibitors (perifosine **Figure 1**, and MK-2206 **Figure 3**), to further investigate whether ATXN3 had the similar effect when the cells were treated with chemotherapeutic drugs, we transfected ATXN3 siRNA #1 and #2 into AS cells, and transfected ATXN3 siRNA#2 into BE2 (marked as ATXN3 siRNA), and then treated the cells with etoposide for 48 h, cell confluence (**Figures 4A, B**), cell survival (**Figures 4C, D**), and cell apoptosis (**Figures 4E, F**) were evaluated by using similar assays described in **Figure 1**.

Under the condition of etoposide treatment, different from perifosine and MK-2206, the confluence curves of the cells transfected with ATXN3 siRNAs (purple curve) were significantly higher than that of the cells transfected with control siRNA (green curve) (**Figures 4A, B**, left). After a 48-h treatment of etoposide, there was a statistically significant increase of cell confluence in the cells transfected with ATXN3 siRNA compared with the cells transfected with control siRNA (for etoposide treatment, in AS cells, ATXN3 siRNA #1 and #2 *vs.* control siRNA: 83.3% and 74.5% *vs.* 46.2%, P<0.01, **Figure 4A**, right; In BE2 cells, ATXN3 siRNA *vs.* control siRNA: 80.1% *vs.* 58.3%, P<0.01, **Figure 4B**, right).

With etoposide treatment, the survival rate of the cells transfected with ATXN3 siRNAs was significantly higher than that of the cells transfected with control siRNA (for etoposide treatment, in AS cells, ATXN3 siRNA #1 and #2 *vs.* control siRNA: 48.0% and 55.1% *vs.* 22.5%, P<0.01, **Figure 4C**; in BE2 cells, ATXN3 siRNA *vs.* control siRNA: 75.7% *vs.* 56.3%, P<0.01, **Figure 4D**).

For Annexin V/PI flow cytometry, under the condition of etoposide treatment, the apoptotic rate of NB cells with ATXN3 siRNAs transfected was significantly lower than the control group (with etoposide treatment, in AS cells, ATXN3 siRNA #1 and #2 *vs.* control siRNA: 24.1% and 27.7% *vs.* 38.4%, P<0.001, **Figure 4E**, right; in BE2 cells, ATXN3 siRNA *vs.* control siRNA: 19.1% *vs.* 28.8%, P<0.01, **Figure 4F**, right). Besides, under microscope, there were fewer dead cells and more living cells when AS and BE2 cells were treated with a combination of ATXN3 downregulation and etoposide treatment compared to those treated with etoposide treatment only (**Figure 4G**). These results suggested that downregulation of ATXN3 significantly inhibits etoposide-induced cell death.

### Downregulation of ATXN3 Decreased the Sensitivity of NB Cells to Cisplatin

**Figure 5:**
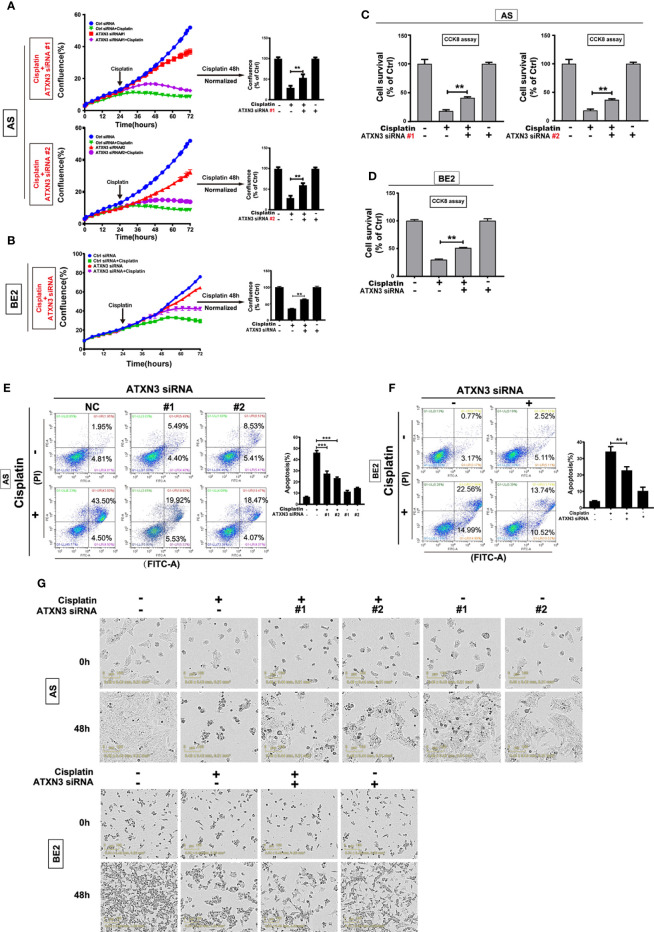
Downregulation of ATXN3 decreased the sensitivity of NB cells to cisplatin. AS cells were transfected with ATXN3 siRNAs (#1, #2) and BE2 cells were transfected with ATXN3 siRNA #2 (marked as ATXN3 siRNA) for 16 h, followed by 48 h treatment of cisplatin. **(A, B)** Cell confluence was dynamically detected and analyzed by IncuCyte Zoom; **(C, D)** Cell survival was detected by CCK8 assay; **(E, F)** Cell apoptosis was detected by Annexin V/PI flow cytometry; **(G)** The images of AS and BE2 cells with ATXN3 siRNAs transfection and cisplatin treatment at 0 h and 48 h were recorded. Bar, SD, ***, P<0.01, **, P<0.01, ATXN3 siRNAs +cisplatin *vs.* control siRNA + cisplatin.

As downregulation of ATXN3 decreased the sensitivity to etoposide in AS and BE2 cells (**Figure 4**), that was different from the promoting effect of ATXN3 downregulation in the cell death induced by AKT inhibitors (**Figure 1** for perifosine, **Figure 3** for MK-2206), we investigated the role of ATXN3 in another chemotherapeutic drug, cisplatin, which is commonly used for the treatment of NB patients. We transfected ATXN3 siRNAs #1 and #2 into the AS cells and ATXN3 siRNA#2 (marked as ATXN3 siRNA) into BE2 cells, and then treated the cells with cisplatin for 48 h, cell confluence (**Figures 5A, B**), cell survival (**Figures 5C, D**), and cell apoptosis (**Figures 5E, F**) were evaluated by using similar assays described in **Figure 1**.

Under the condition of cisplatin treatment, the confluence curves of the cells transfected with ATXN3 siRNAs (purple curve) were significantly higher than that of control siRNA (green curve) (**Figures 5A, B**, left). Forty-eight hours after cisplatin treatment, there was a statistically significant increase of cell confluence in the cells transfected with ATXN3 siRNAs compared to the cells transfected with control siRNA (For cisplatin treatment, in AS cells, ATXN3 siRNA #1 and #2 *vs.* control siRNA: 53.6% and 60.3% *vs.* 29.4%, P<0.01, **Figure 5A**, right; in BE2 cells, ATXN3 siRNA *vs.* control siRNA: 63.3% *vs.* 35.2%, P<0.01, **Figure 5B**, right).

Under the condition of cisplatin treatment, the survival rates of the cells in ATXN3 siRNAs were statistically higher than that of the cells transfected with control siRNA (For cisplatin treatment, in AS cells, ATXN3 siRNA #1 and #2 *vs.* control siRNA: 41.2% and 36.8% *vs.* 18.7%, P<0.01, **Figure 5C**; in BE2 cells, ATXN3 siRNA *vs.* control siRNA: 51.1% *vs.* 30.0%, P<0.01, **Figure 5D**).

For Annexin V/PI flow cytometry, under the condition of cisplatin treatment, the apoptotic rate of NB cells with ATXN3 siRNAs transfected was significantly lower than control siRNA group (For cisplatin treatment, in AS cells, ATXN3 siRNA #1 and #2 *vs.* control siRNA: 27.4% and 23.3% *vs.* 46.1%, P<0.001, **Figure 5E**, right; in BE2 cells, ATXN3 siRNA *vs.* control siRNA: 22.8% *vs.* 34.2%, P<0.01, **Figure 5F**, right). Besides, under microscope, there were fewer dead cells and more living cells when AS and BE2 cells were treated with a combination of ATXN3 downregulation and cisplatin compared to those treated with cisplatin only (**Figure 5G**). These results suggested that downregulation of ATXN3 significantly inhibit cisplatin-induced cell death in NB cells.

### Downregulation of ATXN3 Decreased the Sensitivity of NB Cells to Etoposide and Cisplatin *via* Upregulating Bcl-xl

**Figure 6:**
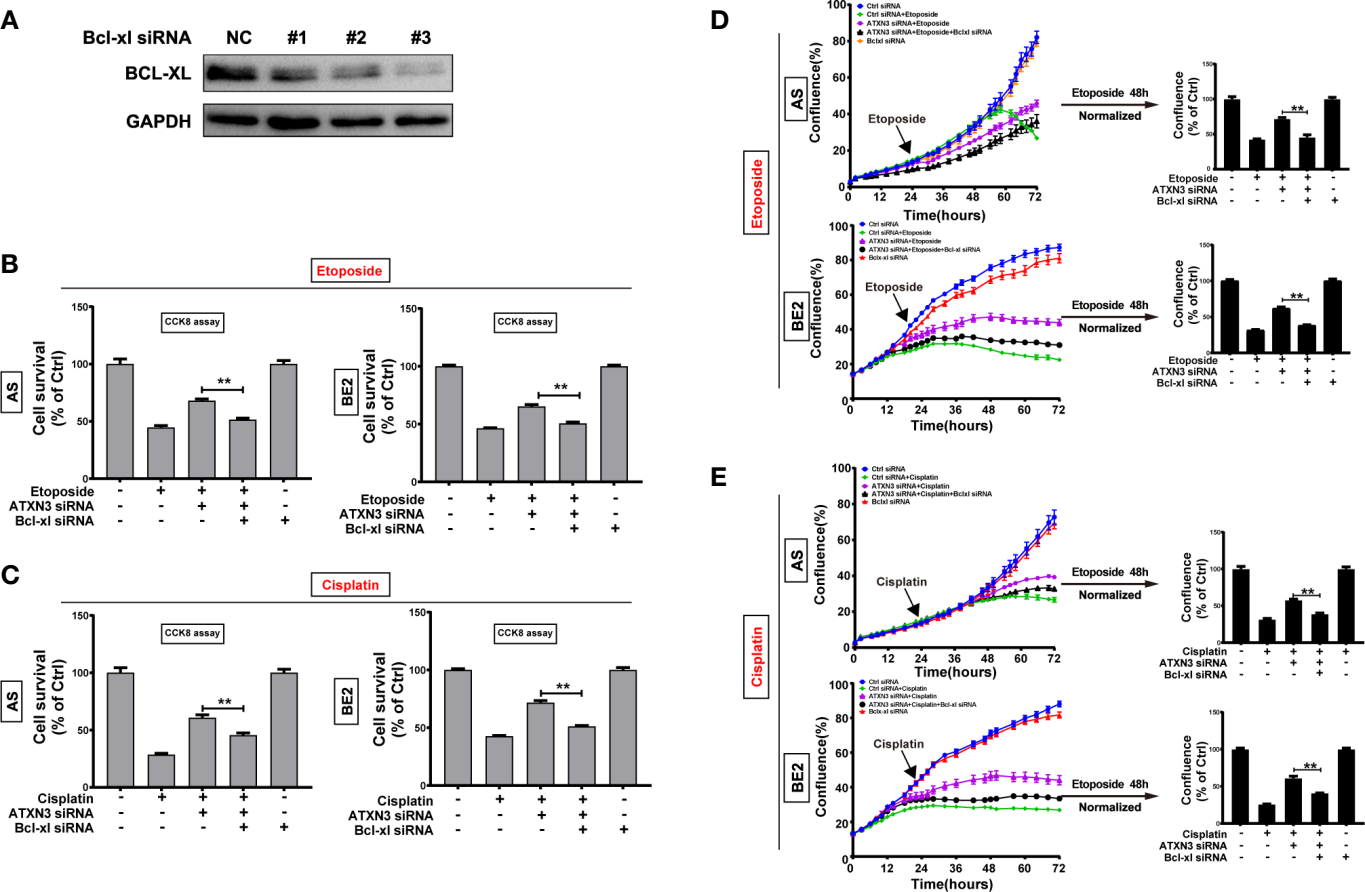
Silencing Bcl-xl attenuated the decrease in sensitivity to etoposide and cisplatin caused by the downregulation of ATXN3. **(A)** AS and BE2 cells were transfected with Bcl-xl siRNAs (#1, #2, and #3) for 48 h, and the expression of Bcl-xl was detected by western blot; **(B, C)** Bcl-xl siRNAs#3(marked as Bcl-xl siRNA) and ATXN3 #2(marked as ATXN3 siRNA) were transfected into AS and BE2 cells alone or combination, then the cells were treated with etoposide (AS:1μg/ml; BE2: 4μg/ml) or cisplatin (AS:1μg/ml; BE: 2μg/ml) for 48 h, cell survival was detected by CCK8 assay; **(D, E)** the confluence of etoposide was detected and analyzed by IncuCyte Zoom. Bar, SD, **P < 0.01, ATXN3 siRNAs + etoposide/cisplatin + Bcl-xl siRNA *vs.* ATXN3 siRNA + etoposide/cisplatin.

As we found that downregulation of ATXN3 increased the expression of Bcl-xl significantly (as shown in **Figure 2A**), to investigate whether Bcl-xl mediates the process that downregulation of ATXN3 decreased the sensitivity of NB cells to etoposide and cisplatin, three Bcl-xl siRNAs (#1, #2, and #3) were designed and evaluated, and the Bcl-xl siRNA #3 decreased the expression of Bcl-xl significantly (**Figure 6A**). Then we transfected the ATXN3 siRNA #2 (marked as ATXN3 siRNA) and Bcl-xl siRNA #3 (marked as Bcl-xl siRNA) into AS and BE2 cells, and then treated them with etoposide or cisplatin for 48 h. Then, cell survival (**Figures 6B, C**) and cell confluence (**Figures 6D, E**) were evaluated by CCK8 assay and IncuCyte Zoom machine respectively.

The cell survival analysis detected by CCK8 assay showed that the survival rate of cells treated with etoposide/cisplatin +ATXN3 siRNA + Bcl-xl siRNA was significantly lower than that of the cells treated with etoposide/cisplatin + ATXN3 siRNA (for etoposide treatment, ATXN3 siRNA + Bcl-xl siRNA *vs.* ATXN3 siRNA, in AS cells: 51.5% *vs.* 68.0%, P<0.01, **Figure 6B**, left; in BE2 cells, 50.6% *vs.* 65.3%, P<0.01, **Figure 6B**, right; for cisplatin treatment, in AS cells: 45.6% *vs.* 60.7%, P<0.01, **Figure 6C**, left; in BE2 cells: 51.2% *vs.* 71.7%, P<0.01, **Figure 6C**, right). Also, the confluence curve of the cells treated with etoposide/cisplatin +ATXN3 siRNA + Bcl-xl siRNA (black curve) was significantly lower than that of the cells treated with etoposide/cisplatin + ATXN3 siRNA (purple curve) (**Figures 6D, E**, left). Forty-eight hours after etoposide or cisplatin treatment, there was a statistically significant decrease of cell confluence in NB cells transfected with ATXN3 siRNA + Bcl-xl siRNA compared with cells transfected with ATXN3 siRNA only(ATXN3 siRNA + Bcl-xl siRNA *vs.* ATXN3 siRNA, for etoposide treatment, in AS cells: 45.4% *vs.* 71.8%, P<0.01; in BE2 cells: 38.2% *vs.* 61.8%, P<0.01, **Figure 6D**, right; for cisplatin treatment, in AS cells: 38.9% *vs.* 57.7%, P<0.01; in BE2 cells: 40.9% *vs.* 61.0%, P<0.01, **Figure 6E**, right). These results indicated that downregulation of Bcl-xl partially attenuated the effect of the downregulation of ATXN3 decreased the sensitivity of NB cells to chemotherapeutic drugs (etoposide and cisplatin).

## Discussion

In this study, we found that perifosine or MK-2206 increased the expression of ATXN3 in AS cells. Downregulation of ATXN3 significantly enhanced the cell death induced by AKT inhibitors (perifosine or MK-2206) *via* upregulating BIM. Although different from AKT inhibitors, downregulation of ATXN3 decreased the sensitivity of NB cells to chemotherapeutic drugs (etoposide or cisplatin), and Bcl-xl mediates this process.

As a member of deubiquitylates, ATXN3 mainly involved in the maintenance of protein homeostasis, and degradation of misfolded substrate and cell death (40–43). Over decades, researches mainly focused on the molecular mechanism underlying Machado-Joseph disease (SCA3) caused by the aggression of ATXN3 (44). Recently, studies have shown that ATXN3 plays a vital role in tumorigenesis (35, 36, 45). Zou et al. found that ATXN3 binds to KLF4, and high expression of ATXN3 promoted breast cancer metastasis by deubiquitinating and stabilizing KLF4 (35). Rodrigues et al. found that absence of ATXN3 increases cell death by reducing the metabolic activity in HeLa cells (46). Also, Gao et al. reported that mutant ATXN3 could trigger apoptosis in SH-SY5Y cells *via* activating P53 (47).

AKT is a key regulator of PI3K/AKT/mTOR pathway, and its abnormal activation can significantly promote progression of tumor, it is also an important target for clinical treatment (48, 49). Daniela et al. found that the activation of AKT is an independent prognostic factor and significantly correlated with MYCN amplification and advanced stage in a study with 116 NB patients (12), suggesting that AKT may be an important potential target for NB treatment. Perifosine and MK-2206 are AKT inhibitors with potential anti-tumor effects, as supported by the phase II clinical trials of perifosine in head and neck cancer (50), melanoma (51), breast cancer (52), and colorectal cancer (53), prostate cancer (54) and cervical cancer (55) showing that perifosine has satisfactory therapeutic effects. In a multicenter phase I clinical trial of perifosine in the treatment of recurrent refractory NB, perifosine showed low toxicity, well tolerance, and as a single agent, perifosine prolonged the survival time of patients significantly (20, 56). Also, MK-2206 also showed good anti-tumor effects in clinical trials in lymphocytic leukemia (57), breast cancer (58), endometrial carcinoma (58), non-small cell lung cancer (59), pancreatic cancer (60) and prostate cancer (61). In our previous studies, we reported that AKT inhibitors perifosine or MK-2206 protected against tumor growth *in vitro* and *in vivo* as a single agent, and increased the therapeutic effects of chemotherapeutic drugs in NB (17–19).

The sensitivity of perifosine and MK-2206 is affected by many factors, Simons et al. found that the inhibition of glutathione and thioredoxin metabolism could decrease the cell death induced by perifosine in head and neck cancer (62). Furthermore, blocking autophagy could increase the sensitivity of lung cancer cells (23) and glioma cells (63) to perifosine, and increase MK-2206-induced cell death of melanoma (64). Ashkenazi et al. found that ATXN3 interacts with Beclin directly, and regulates beclin 1-dependent autophagy (65). Besides, Sacco et al. found that downregulation of ATXN3 could inhibit the activation of AKT and decrease the cell viability by inducing the expression of PTEN in lung cancer (36). The downregulation of ATXN3 could also inhibit the proliferation of testicular cancer cells by inhibiting AKT activation (38). In this study, we found that downregulation of ATXN3 could enhance the cell death induced by AKT inhibitors (perifosine or MK-2206) in NB cells, on one hand, ATXN3 could regulate the activation of AKT, on the other hand, both of perifosine and MK-2206 are AKT inhibitors, thus, downregulation of ATXN3 could increase the sensitivity of NB cells to these agents. Besides, perifosine and MK-2206 could induce the expression of BIM, and promote the apoptosis of cancer cells (66–68). In this study, we found downregulation of ATXN3 promote perifosine and MK-2206 induced the cell death of NB cells *via* upregulating the expression of BIM, which was consistence with those studies mentioned above.

Furthermore, etoposide and cisplatin are first-line chemotherapy drugs for NB patients in clinic (69, 70), and chemotherapy resistance was the most common reason for the failure of NB treatment. Many studies have shown that Bcl-xl could block etoposide induced cell death, and play a very important role in multidrug resistance (71–73). Lebedeva et al. reported that the upregulation of Bcl-xl could inhibit etoposide induced cell death, while downregulation of Bcl-xl could increase the sensitivity of prostate cancer cells to chemotherapeutic agents including etoposide (74). Marengo et al. found that HTLA-ER cells (etoposide resistant NB cells) could alter the BAX/Bcl-xl ratio to avoid apoptosis caused by etoposide (75). Williams et al. reported that over 61.5% of ovarian cancer patients with cisplatin resistance showing Bcl-xl high expression, and high expression of Bcl-xl was resistant to cisplatin in the mouse xenograft model (76). Zhou et al. reported that ATXN3 could interact with Bcl-xl directly in Spinocerebellar ataxia type 3 disease (43). Chou et al. reported that downregulation of ATXN3 could inhibit mitochondrial apoptotic pathway by increasing Bcl-xl in Machado-Joseph disease (77). In this study, we found downregulation of ATXN3 could inhibit apoptosis in NB cells induced by etoposide and cisplatin, leading to a decrease in the sensitivity of NB cells to etoposide and cisplatin. We also found that downregulation of ATXN3 could increase the expression of Bcl-xl. Dole et al. reported that Bcl-xl is highly expressed in NB cells and could inhibit apoptosis induced by etoposide and cisplatin (78). Hadjidaniel et al. found that the upregulation of Bcl-xl is closely related to protection against etoposide and cisplatin induced mitochondrial-dependent apoptosis in NB cells (79). The downregulation of Bcl-xl could reverse cisplatin resistance in ovarian cancer cells, and induce cell death caused by cisplatin at a low concentration (80), and confers sensitivity to cisplatin-resistant mesothelioma cells (81). In this study, we also found that knockdown of Bcl-xl restored the cell death of NB cells with ATXN3 downregulation and etoposide or cisplatin treatment. However, Zhu et al. reported that breast cancer patients with high expression of ATXN3 is correlated with poor prognosis, and downregulation of ATXN3 could increase the sensitivity of breast cancer cells to chemotherapeutic agents (Adriamycin) (45). Differing from the study of Zhu et al, in our present study we used two different chemotherapeutic drugs (etoposide and cisplatin) and NB cells. Studies showed that BIM could bind to Bcl-xl directly by the BH3 domain binding groove (82, 83), and inhibit the function of Bcl-xl (66). In our present study, we found that the downregulation of ATXN3 in NB cells could increase the expression of both BIM and Bcl-xl. It was reported that AKT inhibitors (perifosine or MK-2206) could promote the BIM expression, but decrease Bcl-xl expression (66, 67, 84, 85). So, when the ATXN3-down-regulated NB cells were treated with AKT inhibitors (perifosine or MK-2206), the balance between BIM and Bcl-xl may tilt towards BIM, the accumulation of pro-apoptotic BIM may induce more cell death. While it was reported that etoposide or cisplatin could promote BIM degradation (86, 87). So, when the ATXN3-downregulated NB cells were treated with etoposide or cisplatin, the balance between BIM and Bcl-xl may tilt towards Bcl-xl, and more Bcl-xl would protect NB cell from etoposide or cisplatin-induced cell death.

Our findings in the present study may provide a potential guidance for the selection of treatment regimen. Patients with low ATXN3 expression maybe more sensitive to AKT inhibitors, such as perifosine and MK-2206, and those with high ATXN3 expression may be more suitable for chemotherapeutic drugs, such as etoposide and cisplatin. However, our research still has some limitations, ATXN3 may regulate the sensitivity of NB cells to perifosine and MK-2206 by regulating autophagy. Besides, up to now, this study was only in NB cells, and we will explore the function of ATXN3 in NB xenograft model systematically and comprehensively. Also, we will focus on these questions in our future studies.

In conclusion, downregulation of ATXN3 promoted the cell death of NB cells induced by AKT inhibitors (perifosine and MK-2206) *via* upregulation of BIM, whereas downregulation of ATNX3 did not enhance, but decreased sensitivity of NB cells to chemotherapeutic drugs (etoposide and cisplatin) by upregulating the expression of Bcl-xl in NB cells. These findings suggested that ATXN3 may be a novel potential target for NB therapy and showed great value in providing guidance in precision therapy and medication selection during the treatment of NB patients.

## Data Availability Statement

The original contributions presented in the study are available on request to the corresponding author.

## Author Contributions

BG contributed to the study design, experiment, data analysis, and writing - original daft. ZH and JZ contributed to data collection and analysis. ZLiu and CT contributed to revise the manuscript. ZLi contributed to study design, supervision, project administration, and review and editing of manuscript. All authors contributed to the article and approved the submitted version.

## Funding

This study was supported by National Natural Science Foundation of China (no. 81472359, 81972515), Key Research and Development Foundation of Liaoning province (2019JH8/10300024), 2013 Liaoning Climbing Scholar Foundation, 345 Talent Project of Shengjing Hospital of China Medical University. ZLiu and CT are supported by Center for Cancer Research at the National Institutes of Health in the Intramural Research Program at the NIH.

## Conflict of Interest

The authors declare that the research was conducted in the absence of any commercial or financial relationships that could be construed as a potential conflict of interest.
