# Corrigendum: Downregulation of ATXN3 enhances the sensitivity to AKT inhibitors (Perifosine or MK-2206), but decreases the sensitivity to chemotherapeutic drugs (etoposide or cisplatin) in neuroblastoma cells

**DOI:** 10.3389/fonc.2022.984514

**Published:** 2022-08-03

**Authors:** Baocheng Gong, Jinhua Zhang, Zhongyan Hua, Zhihui Liu, Carol J. Thiele, Zhijie Li

**Affiliations:** ^1^ Department of Pediatrics, Shengjing Hospital of China Medical University, Shenyang, China; ^2^ Medical Research Center, Liaoning Key Laboratory of Research and Application of Animal Models for Environment and Metabolic Diseases, Shengjing Hospital of China Medical University, Shenyang, China; ^3^ Cellular and Molecular Biology Section, Pediatric Oncology Branch, National Cancer Institute, National Institutes of Health, Bethesda, MD, United States

**Keywords:** neuroblastoma, ataxin-3, BIM, Bcl-xl, perifosine, MK-2206, etoposide, cisplatin

In the original article, there were mistakes in **Figure 1**, **Figure 3**, **Figure 4** and **Figure 5** as published. The mistakes are in **Figure 1A** **-**right (GAPDH), **Figure 3H** **-**right (GAPDH), **Figure 4G** (BE2 cells: 48h for Ctrl siRNA + Etoposide) and **Figure 5G** (AS cells: 0h and 48h for ATXN3 siRNA#2), as these images were misused. The corrected **Figure 1**, **Figure 3**, **Figure 4** and **Figure 5** appear below.

**Figure 1:**
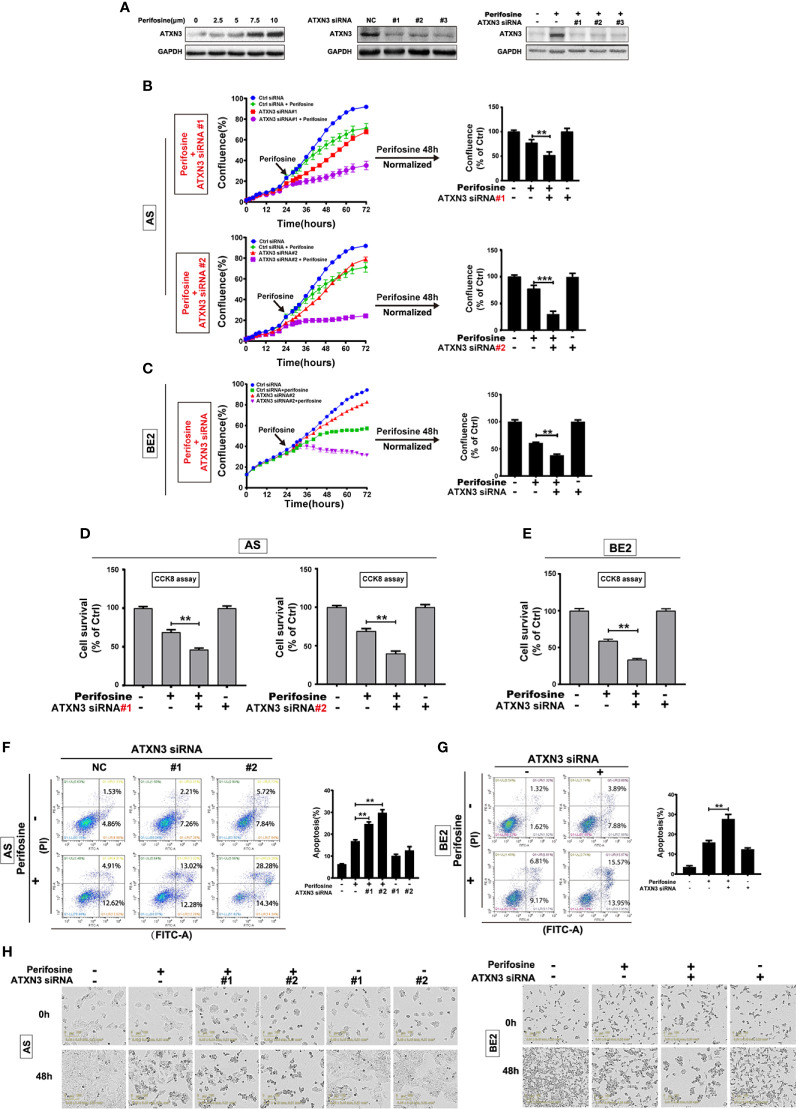
Downregulation of ATXN3 promoted perifosine-induced cell death in NB cells. AS cells were treated with different concentrations of perifosine (2.5, 5, 7.5, and 10 μM) for 24 h, or transfected with ATXN3 siRNA (#1, #2, #3, and control) for 48 h, or transfected with ATXN3 siRNA (#1, #2, and #3) for 16 h followed by 24 h treatment of perifosine. **(A)** The expression of ATXN3 were detected by Western blot; **(B, C)** AS cells were transfected with ATXN3 siRNAs (#1, #2), and BE2 cells were transfected with ATXN3 siRNA2 (marked as ATXN3 siRNA) for 16 h, followed by 48 h treatment of perifosine: Cell confluence was dynamically detected by IncuCyte Zoom and analyzed at the end of experiment; **(D, E)** Cell survival was detected by CCK8 assay; **(F, G)** cell apoptosis was detected by Annexin V/PI flow cytometry; **(H)** The images of AS and BE2 cells with ATXN3 siRNAs transfection and perifosine treatment at 0 h and 48 h were recorded. Bar, SD, **P < 0.01, ATXN3 siRNAs + perifosine vs. control siRNA + perifosine; ***P < 0.001, ATXN3 siRNA #2 + perfosine vs. control siRNA + perifosine.

**Figure 3:**
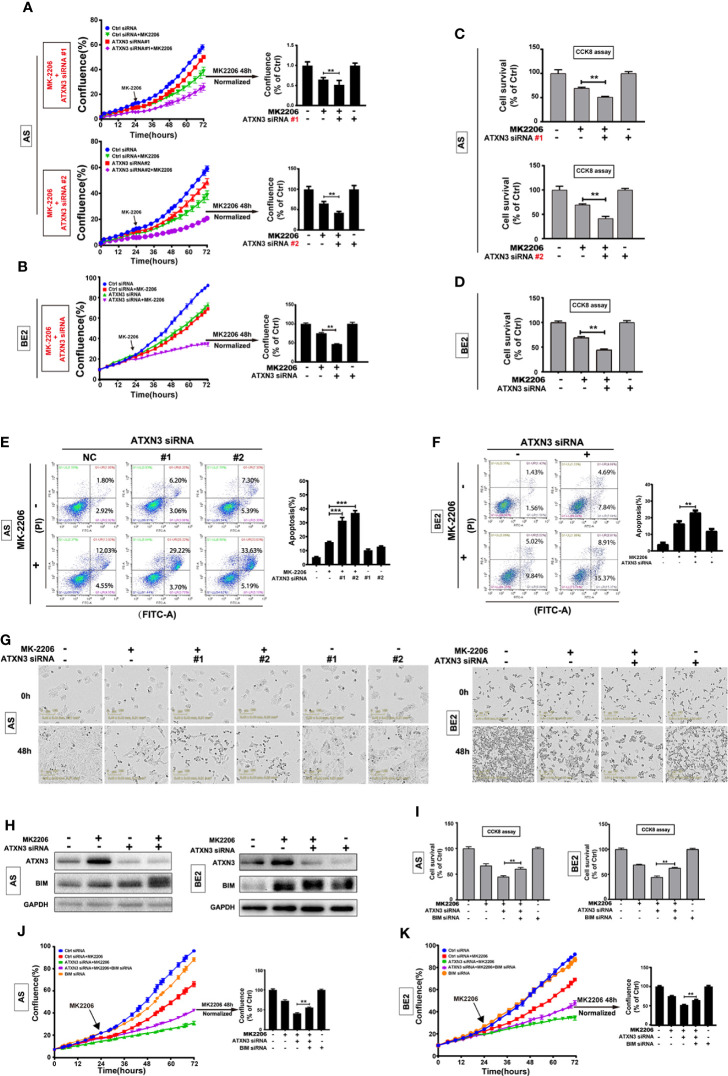
BIM mediated the cell death induced by a combination of MK-2206 treatment and ATXN3 downregulation in NB cells. AS cells were transfected with ATXN3 siRNAs (#1 and #2) and BE2 cells were transfected with ATXN3 siRNA #2 (marked as ATXN3 siRNA) for 16 h followed by 48 h treatment of MK-2206. **(A, B)** Cell confluence was dynamically detected by IncuCyte Zoom and analyzed at the end of experiment; **(C, D)** Cell survival was detected by CCK8 assay; **(E, F)** Cell apoptosis was detected by Annexin V/PI flow cytometry. Bar, SD, **, P< 0.01, ***, P<0.001, ATXN3 siRNAs + perifosine vs. control siRNA + perifosine; **(G)** The images of AS and BE2 cells with ATXN3 siRNAs transfection and perifosine treatment at 0 and 48 h were recorded; **(H)** AS and BE2 cells were transfected with ATXN3 siRNA2 (marked as ATXN3 siRNA) for 16 h, and treated with MK-2206 for 24 h, the expression of ATXN3 and BIM was detected by Western blot; **(I)** BIM siRNA #1 (marked as BIM siRNA) and ATXN3 siRNA #2 (marked as ATXN3 siRNA) were transfected into AS and BE2 cells alone or combination, then the cells were treated with MK-2206 for 48 h, cell survival was detected by CCK8 assay; **(J, K)** Cell confluence was detected by IncuCyte Zoom. Bar, SD, **, P<0.01, ATXN3 siRNA + MK-2206 + BIM siRNA vs. ATXN3 siRNA + MK-2206.

**Figure 4:**
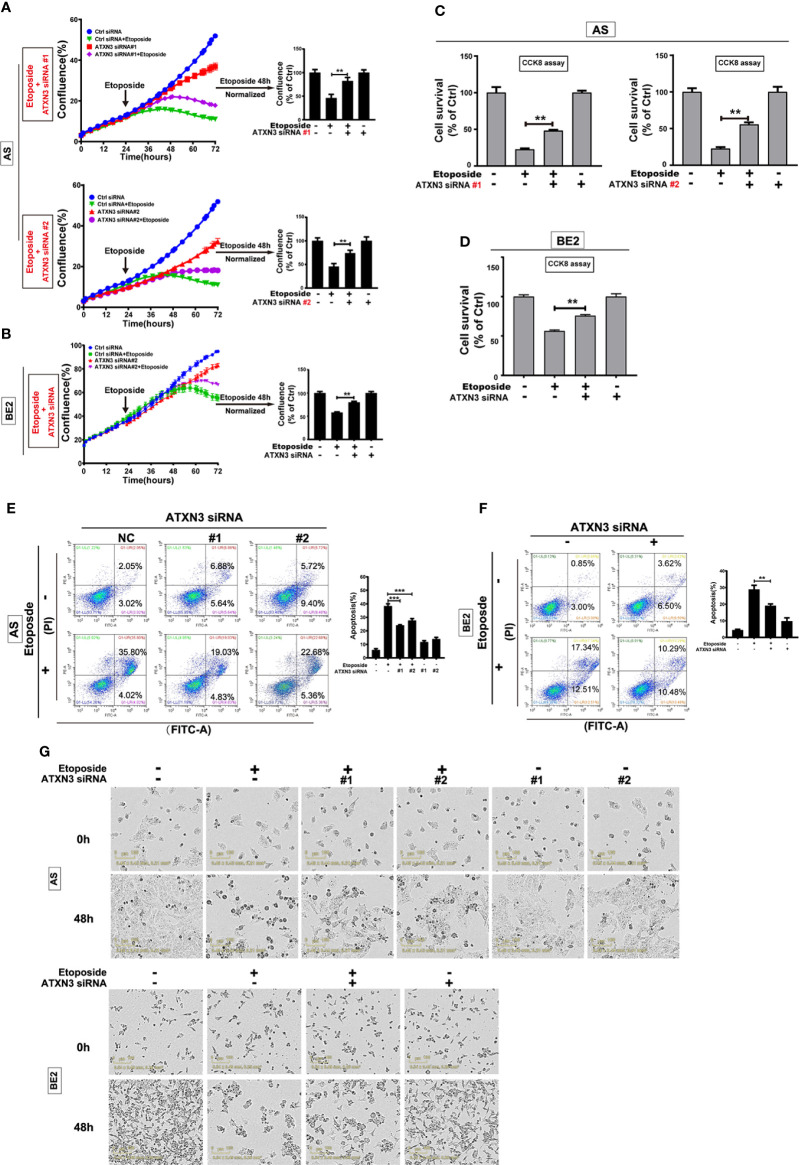
Downregulation of ATXN3 decreased the sensitivity of NB cells to etoposide. AS cells were transfected with ATXN3 siRNAs (#1, #2) and BE2 cells were transfected with ATXN3 siRNA #2 (marked as ATXN3 siRNA) for 16 h, followed by 48 h treatment of etoposide. **(A, B)** Cell confluence was dynamically detected and analyzed by IncuCyte Zoom; **(C, D)** Cell survival was detected by CCK8 assay; **(E, F)** Cell apoptosis was detected by Annexin V/PI flow cytometry; **(G)** The images of AS and BE2 cells with ATXN3 siRNAs transfection and etoposide treatment at 0 h and 48 h were recorded. Bar, SD, ***, P<0.001, **, P<0.01, ATXN3 siRNAs + etoposide vs. control siRNA + etoposide.

**Figure 5:**
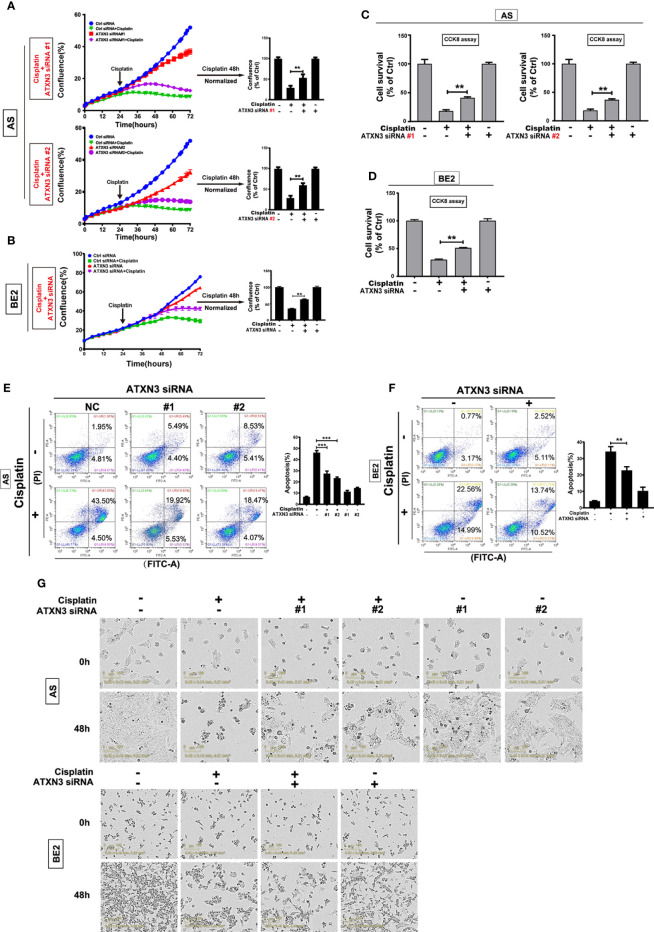
Downregulation of ATXN3 decreased the sensitivity of NB cells to cisplatin. AS cells were transfected with ATXN3 siRNAs (#1, #2) and BE2 cells were transfected with ATXN3 siRNA #2 (marked as ATXN3 siRNA) for 16 h, followed by 48 h treatment of cisplatin. **(A, B)** Cell confluence was dynamically detected and analyzed by IncuCyte Zoom; **(C, D)** Cell survival was detected by CCK8 assay; **(E, F)** Cell apoptosis was detected by Annexin V/PI flow cytometry; **(G)** The images of AS and BE2 cells with ATXN3 siRNAs transfection and cisplatin treatment at 0 h and 48 h were recorded. Bar, SD, ***, P<0.01, **, P<0.01, ATXN3 siRNAs + cisplatin vs. control siRNA + cisplatin.

The authors apologize for this error and state that this does not change the scientific conclusions of the article in any way. The original article has been updated.

## Publisher’s note

All claims expressed in this article are solely those of the authors and do not necessarily represent those of their affiliated organizations, or those of the publisher, the editors and the reviewers. Any product that may be evaluated in this article, or claim that may be made by its manufacturer, is not guaranteed or endorsed by the publisher.

